# Giant retroperitoneal lymphangioma in a 70-year-old male: a case report

**DOI:** 10.11604/pamj.2022.42.153.34175

**Published:** 2022-06-24

**Authors:** Aymen Mabrouk, Farouk Ennaceur, Yasser Karoui, Eya Ben Nejma, Laila Jedidi, Mounir Ben Moussa

**Affiliations:** 1 Department of General Surgery A, University Hospital Charles Nicolle, Faculty of Medicine of Tunis, University of Tunis El Manar, Tunis, Tunisia

**Keywords:** Cystic lymphangioma, retro-peritoneal cyst, case report

## Abstract

Lymphangiomas are relatively rare benign congenital tumors of the lymphatic system. They are usually discovered during childhood and typically occur in the neck and axillary regions. Retroperitoneal lymphangiomas are a rare occurrence and represent 1% of all cases. Here, we presented a 70-year-old male who presented with abdominal discomfort and chronic constipation in the last three years. A computerized tomography scan showed a giant abdominal hypodense cystic mass measuring 195 x 145 mm, which laminates the abdominal aorta, the left iliac vessels, and the left ureter. The patient underwent surgical exploration. Due to the giant cystic volume, it was carefully opened, aspirated, and removed. The histopathological examination showed a retroperitoneal lymphangioma. In conclusion, giant retroperitoneal lymphangioma in an adult is a rare occurrence. The primary treatment is complete surgical excision. Histopathological examination is essential for diagnosis confirmation.

## Introduction

Lymphangiomas are relatively rare benign congenital tumors of the lymphatic system. Usually discovered in children and localized in the cervical and axillary regions [1]. Retroperitoneal lymphangiomas represent only 1% of all localizations. Clinical manifestations of lymphangiomas are not specific, such as vague pain, bowel obstruction, and abdominal mass [1,2]. It can be revealed in very rare cases by complications such as hemorrhage, infection, or rupture [1]. Computed Tomography (CT) scan is helpful for the diagnosis. However, the final diagnosis is usually obtained by histopathological examination of the specimen. Surgical complete monobloc resection is the best treatment, with a low recurrence rate [1]. In adults, giant lymphangiomas with retroperitoneal origin are rarely reported in the literature. Hence, we represent a giant retroperitoneal lymphangioma case in a 70-year-old male patient.

## Patient and observation

**Patient information:** a 70-year-old male with a medical history of hypertension and diabetes presented with abdominal distention and chronic constipation, evolving for three years with no other associated symptoms. The patient had no other symptoms such as vomiting, abdominal pain, or urinary tract symptoms.

**Clinical findings:** on examination, the patient was afebrile with a good general condition. The vital signs were temperature: 37.3°C, blood pressure: 14/07 mmHg, and pulse rate: 90 bpm. Abdominal examination revealed a fixed painless renitent mass in the left lower quadrant of the abdomen measuring about 15 cm.

**Figure 1:**
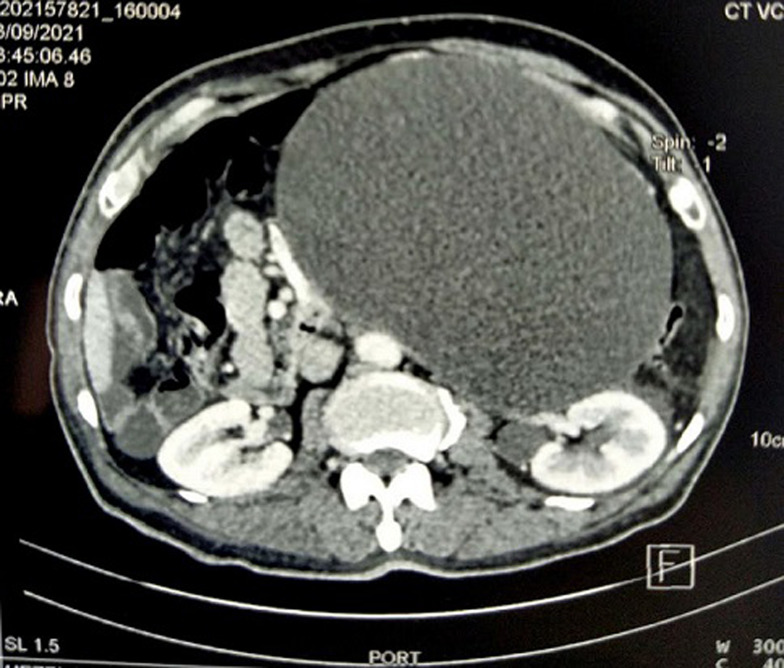
computerized tomography scan showing a huge intraperitoneal hypodense cystic mass measuring 195 x 145 mm in width and 245 mm in length that occupied the aorta, the left iliac vessels, and the left ureter

**Diagnostic assessment:** laboratory tests showed white blood cells were 10 x 10^3^/ml, C-reactive protein was 100 mg/dL, hydatid serology and tumor markers were negative. A CT scan showed a huge intraperitoneal hypodense cystic mass of the left abdomen, thin-walled with no proper vascularization, measuring 195 x 145 mm in width and 245 mm in length. It laminates the abdominal aorta, the left iliac vessels, and the left ureter with no adjacent organ invasion without satellite lymphadenopathies (Figure 1).

**Diagnosis:** the main differential diagnosis is mesenchymal tumors, germ cell tumors, and hydatid cysts since echinococcosis infections are endemic in our country; therefore, a fine needle aspiration was not an option.

**Figure 2:**
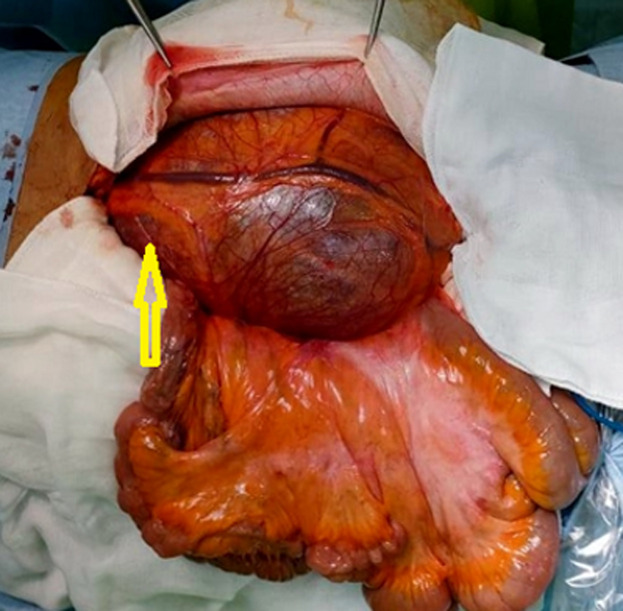
intraoperative photo showing the mass (arrow)

**Figure 3:**
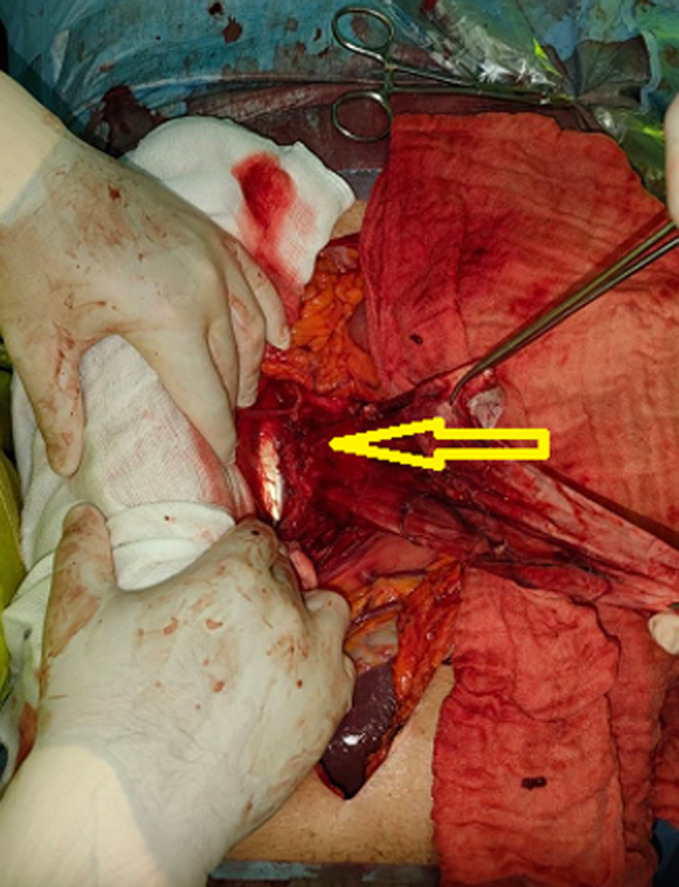
intraoperative photo showing the abdomen after cyst resection (arrow); the mass adhered to the left ureter, the abdominal aorta, and the left psoas muscle

**Therapeutic interventions:** the decision was to perform a surgical resection. The patient underwent a midline laparotomy. The cyst was found in the retroperitoneum on the left side and measured 20 x 15 cm (Figure 2). A monobloc resection was hazardous due to the volume of the cyst. The cyst was carefully opened, and its content was aspirated (1500 ml). Total cystectomy was carefully performed since the adhesions with the left ureter, the abdominal aorta, and the left psoas muscle (Figure 3). Surgical exploration revealed a 1.5 cm mass of the cephalic pancreas that was easily enucleated.

**Figure 4:**
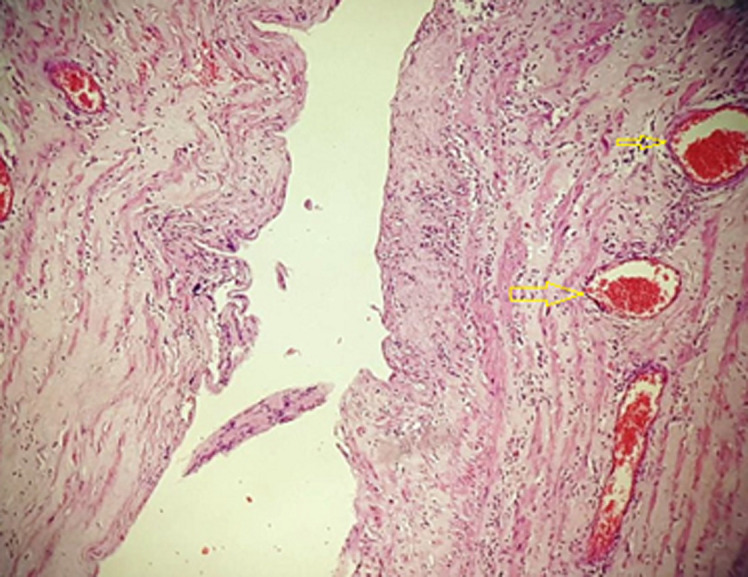
histological image shows that the muscular wall cyst is lined by a single layer of benign, flattened endothelium (arrows)

**Follow-up and outcome of interventions:** the operation was complicated by a pancreatic fistula discovered on 20 postoperative days and was treated with Sansdostatine injection, radio-guided drainage, and endoscopic sphincterotomy. The patient was discharged after 27 days without complications. The histopathological examination of the specimens showed large dilated cysts lined by flattened endothelial cells, suggestive of cystic lymphangioma and a benign cystadenoma of the pancreas (Figure 4). A 6 months outpatient follow-up didn´t reveal any abnormalities.

**Patient perspective:** according to the patient, his experience with his disease was unexpected. Symptoms that were not intense involved diagnostic testing and surgical treatment, with an extended follow-up. At the end of the process, he is satisfied.

**Informed consent:** written informed consent was obtained from the patient for participation in our study.

## Discussion

Lymphangioma is a rare, benign fluid-filled cystic anomaly originating from lymphatic vessels with an excellent prognosis. It frequently appears in the head and neck, with a high prevalence in children and young adults and equivalent in males and females [1,2]. Lymphangioma is assumed to be congenital, resulting from lymphatic sequestrations that do not connect with the normal lymphatic channels. Lymphangioma is also thought to be caused by fibrosis, trauma, and neoplasms [3]. Cystic lymphangiomas represent 7% of abdominal cystic lesions in adults, and 95% occur in the neck and axillaries regions. The remaining 5% localizations are the mesentery, retroperitoneum, abdominal viscera, lung, and mediastinum [4]. The retroperitoneal localization represents just 1%. In most cases, they remain asymptomatic but may cause nonspecific manifestations such as vague abdominal pain or discomfort in 75% of cases [5,6]. Similarly, our patient complains of abdominal discomfort.

Lymphangiomas are frequently multilocular cystic masses that are anechoic or contain echogenic debris on sonography. A CT scan with intravenous contrast may enhance the cyst wall and septa. Typically, the fluid component is homogeneous and has low attenuation values. In the presence of chyle, negative attenuation values can occur. Calcification is possible but uncommon. The intra-cystic attenuation values may mimic a solid tumor mass or an abscess if bleeding occurs. Lesions can move through adjacent retroperitoneal anatomical compartments, displacing organs and vessels. They can compress and infiltrate vital structures and cause complications such as intra-cystic hemorrhage, cyst rupture, volvulus, and infection. The sonographic appearance of a septated cystic mass with clear fluid is thought to be a sign of lymphangioma. However, preoperative diagnosis is frequently difficult because there is little to differentiate them from those of other cystic masses. Because of its rarity, the lesion is usually not considered in the differential diagnosis. Cystic lymphangioma is distinguished by an elongated shape and a crossing from one retroperitoneal compartment to another. On CT, cystic lymphangioma appears as a large, thin-walled, multi-septate cystic mass. A teratoma with a considerable cystic component may depict a lymphangioma, as mentioned in our case´s CT scan [6-8].

The primary differential diagnosis of retroperitoneal lymphangiomas is hydatid cyst, mesenchyme tumors, lymphomas, and germ cell tumors [9]. Radical surgical resection (monoblock resection) is the standard treatment for giant lymphangioma tumors, whether by laparoscopy or laparotomy [10]. However, complete resection may be challenging due to vascular and organ adhesions. Some authors suggest nonoperative treatment such as aspiration of contents and injection of sclerosant agents when the dissection is hazardous and has been demonstrated to be effective. In cases with active bleeding, arteriography embolization could be attempted. Hubli *et al*., Khan *et al*., and Tripathi *et al*. have reported similar cases in which complete excision was supported as the preferred treatment [3,11,12]. Radiation therapy is another option. This treatment option is generally inadequate for lymphangiomas, but there has been some success with lymphangiolipoma. On small lymphangioma lesions, cryotherapy and electrocautery have been used. Carbon dioxide laser vaporization and the combination of laser light and radiofrequency energy are effective and relatively safe [13-15]. On histopathological examination, lymphangioma consists of cavities lined by an endothelium resting on fibrous tissue containing lymphocytic islets and sometimes smooth muscle fibers. It can be uni or multi-locular. The content can be chylous, hemorrhagic, or serous. Inflammation and bleeding often cause reshaping with the disappearance of the endothelium and the appearance of fibrin deposits, sometimes making the histological diagnosis impossible on superficial biopsies [16]. The final diagnosis in our case was attempted by histopathological examination of the specimen. The recurrence rates ranged between 1% and 13.6%, and most recurrences occurred with partial resection or retroperitoneal localization [17]. After six months of follow-up, there were no signs of recurrence in our case.

## Conclusion

Retroperitoneal lymphangiomas are a rare adult lesion. Radiological examinations can provide pre-operative diagnosis, but in cases with large cysts and advanced age, the diagnosis can be inconclusive, and the final diagnosis should be attempted by histopathological examination of the specimen, like with our patient. Surgical monoblock resection is a gold standard treatment option with an excellent prognosis, symptomatic relief, and cure.
